# Health Disparities among Patients with Cancer Who Received Molecular Testing for Biomarker-Directed Therapy

**DOI:** 10.1158/2767-9764.CRC-24-0321

**Published:** 2024-10-04

**Authors:** Elisabeth Heath, Gregory Dyson, Jennifer R. Ribeiro, Joanne Xiu, Kelsey Poorman, Hirva Mamdani, Mohammed N. Al-Hallak, Anthony F. Shields, Jailan A. Elayoubi, Ira S. Winer, Frank C. Cackowski, Gary A. Puckrein, Gilberto de Lima Lopes, Nathaniel Jones, Ralph J. Hauke, Samuel A. Kareff, Milan Radovich, George W. Sledge, David B. Spetzler, Gregory A. Vidal, John L. Marshall

**Affiliations:** 1 Department of Oncology, Karmanos Cancer Institute, Wayne State University, Detroit, Michigan.; 2 Caris Life Sciences, Phoenix, Arizona.; 3 National Minority Quality Forum, Washington, District of Columbia.; 4 Division of Medical Oncology, Sylvester Comprehensive Cancer Center, University of Miami Miller School of Medicine, Miami, Florida.; 5 Mitchell Cancer Institute, University of South Alabama, Mobile, Alabama.; 6 Nebraska Cancer Specialists, Omaha, Nebraska.; 7 Caris Life Sciences, Irving, Texas.; 8 West Cancer Center and Research Institute, Germantown, Tennessee.; 9 Division of Hematology and Oncology, MedStar Health/Lombardi Comprehensive Cancer Center Georgetown University, Washington, District of Columbia.

## Abstract

Health disparities present a barrier to successful oncology treatment. The potential for precision oncology to reduce health disparities has not previously been analyzed. We performed a retrospective analysis of 12,627 patients from six major cancer centers whose tumors underwent molecular testing at Caris Life Sciences between 2010 and 2020. Kaplan–Meier and Cox regression were used to describe and analyze overall survival. The molecular and demographic features of the cohort were analyzed by *χ*^2^ and ANOVA tests. Black patients composed 25% of the cohort and White patients 63%. Among this molecularly-tested cohort, there were minimal outcome differences based on race, geographic location, or poverty level. When analyzing the interaction of age, race, and sex, racial-based disparities were noted primarily for young non-White women in the study cohort but were more pronounced for men and women of all ages in the broader patient population within the Surveillance, Epidemiology, and End Results database. Mutations in five genes—*APC*, *EGFR*, *STK11*, *TP53*, and *KRAS*—were found to affect overall survival among our cohort, and their prevalence varied by race in specific tumor types. Real-world outcomes data in mutation-defined cohorts also provided additional context to previously reported therapeutic response trends. Our study shows that patients who undergo molecular testing display reduced racial health disparities compared with the general population, whereas persistent racial disparities are influenced by age and sex. Genomic-driven racial disparities should be examined at a tumor lineage-specific level. Increased access to molecular testing for all eligible patients may play a role in improving health equity.

**Significance:**

This study is the largest of its kind to analyze health disparities and genomic features among a diverse multiinstitutional cohort of patients who underwent molecular testing. Continuing to increase awareness of and access to molecular testing approaches may help to reduce cancer health disparities and improve outcomes for all patients.

## Introduction

Cancer outcomes are affected by multiple variables including age, sex, sexual orientation and gender identity, race, ethnicity, socioeconomic status, geographic location, and insurance access. Preventable differences in outcomes due to these factors are known as cancer health disparities, and present barriers to the goal of equity in healthcare (1, 2). In particular, racial health disparities affecting cancer incidence and mortality have received greater attention in recent years (2). Black males have a higher incidence of cancer overall, and male and female Black patients experience shorter survival relative to White patients for nearly every cancer type (3, 4). Although the racial disparity outcomes gap for all cancers has narrowed since the mid-1990s, the gap has widened or remained steady for certain cancers such as breast and colorectal (3). The continued disparities that exist between Black and White patients stem from a complex interplay of demographic, socioeconomic, and environmental factors, which may affect access to healthcare as well as tumor genomics and biology (3, 5–12).

Despite the possible role of tumor genomics in driving racial cancer disparities, Black and other minoritized populations also have reduced access to genomic testing and clinical trial enrollment for new biomarker-directed therapies (1, 8, 13, 14). Moreover, most large-scale cancer genomic datasets (i.e., AACR-GENIE, The Cancer Genome Atlas, MSK-IMPACT, and UMich Metastatic Solid Cancer) suffer from a lack of diversity representation or fail to report these data at all (1, 15, 16). The implementation of precision oncology has the potential to reduce disparities in cancer outcomes by connecting patients with appropriate therapies and clinical trials; however, the effect of molecular testing implementation on health disparities has not been analyzed in a large real-world cohort. Here, we aimed to understand health disparities among a multicenter cohort of more than 12,000 patients with cancer whose tumors underwent molecular testing at Caris Life Sciences, with a focus on comparing Black and White populations—the two predominant racial groups in the United States. Importantly, Black patients compose 25% of the cohort—the greatest representation of the Black racial group in a large-scale genomic dataset to date—allowing appropriate power to analyze outcomes and molecular correlates associated with race and other demographic characteristics.

## Materials and Methods

### Patient specimens and study sites

A total of 12,627 specimens from six sites underwent molecular testing in a Clinical Laboratory Improvement Amendments/College of American Pathologists–certified laboratory (Caris Life Sciences) between the years 2010 and 2020. Patients’ tumors were profiled as part of their normal care, and demographic information including self-reported race was captured for each patient. All patients whose tumors were profiled were included in the final analysis, with no specific inclusion or exclusion criteria applied. There was no attrition noted. The median age of the cohort was 62.3 years (range: 55.0–71.0 years). The cohort included 52% males/48% females. Tumor enrichment from patient specimens was achieved using manual microdissection techniques prior to DNA and RNA extraction using formalin-fixed, paraffin-embedded kits (Qiagen). The sites included were Alabama (University of South Alabama Mitchell Cancer Institute; *N* = 940), Florida (University of Miami Sylvester Comprehensive Cancer Center; *N* = 553), Michigan (Karmanos Cancer Institute; *N* = 3,225), Nebraska (Nebraska Cancer Specialists; *N* = 946), Tennessee (West Cancer Center; *N* = 4,619), and Washington DC (MedStar Health/Georgetown Lombardi Comprehensive Cancer Center; *N* = 2,344). The Caris CODEai clinico-genomic database, comprising 261,000 patients that are inclusive of patients from the six study sites, was furthermore analyzed for survival trends.

### Molecular testing

Next-generation sequencing was performed on genomic DNA isolated from formalin-fixed, paraffin-embedded tumor samples. Sequencing was performed using Agilent bait panels on Illumina sequencing platforms, per standard Caris protocols for a 592-gene panel (NextSeq) or whole exome sequencing with targeted bait enrichment of 720 clinically relevant genes (NovaSeq 6000). All variants were detected with >99% confidence based on allele frequency and probe panel coverage, with an average sequencing depth of coverage of >500 and an analytic sensitivity of 5%. Genetic variants identified were interpreted by board-certified molecular geneticists and categorized as previously described (17). The copy number alteration of each exon was determined by calculating the average depth of the sample along with the sequencing depth of each exon and comparing this calculated result to a precalibrated value. Gene fusions were detected using the ArcherDx fusion assay and Illumina NovaSeq platform as previously described (18, 19).

### Survival analysis

Overall survival (OS) information was obtained from patient electronic medical records and calculated from the date of tissue collection to the last contact. For Kaplan–Meier analyses performed within the Caris CODEai clinico-genomic database, OS was calculated from tissue collection to last contact and treatment-associated OS from the start of indicated treatment to last contact, based on insurance claims data. Time on treatment (TOT) was calculated from the first day of treatment with the indicated therapy to the last day of treatment with that therapy. Significance was determined as *P* < 0.05.

### Statistical analysis

This was a retrospective analysis of real-world data and therefore not powered to detect specific outcomes. Randomization and blinding were not performed. Statistical methods were employed to determine if health disparities are related to or predictive of patient outcomes in molecularly-tested patients. *χ*^2^ test was used to compare categorical variables and ANOVA to compare continuous variables between groups. Kaplan–Meier and Cox regression were used to describe and analyze OS, respectively. Initial comprehensive Cox regression models were created, and then, nonsignificant variables were eliminated to create a final parsimonious model. The analyses were performed in R software (RRID:SCR_001905).

### Ethical approval

This retrospective repository study was conducted in accordance with the guidelines of the Declaration of Helsinki, the Belmont Report, and the U.S. Common Rule. This study was Institutional Review Board (IRB) approved with waivers of patient written informed consent at each study site (University of South Alabama Mitchell Cancer Institute, University of Miami Sylvester Comprehensive Cancer Center, Karmanos Cancer Institute, Nebraska Cancer Specialists, West Cancer Center, and MedStar Health/Georgetown Lombardi Comprehensive Cancer Center; protocol approval #2016-022). Retrospective analysis of deidentified Caris CODEai data was considered IRB exempt, per 45 CFR 46.101(b)(4), with exempt status determined by Western IRB.

### Data availability

The data presented in this study are not publicly available because of data size and patient privacy, but deidentified data are available for replication and verification purposes on reasonable request. Qualified researchers can apply for access to these data by contacting J. Xiu, PhD, at jxiu@carisls.com and signing a data usage agreement.

## Results

### Cohort characteristics

**Figure 1:**
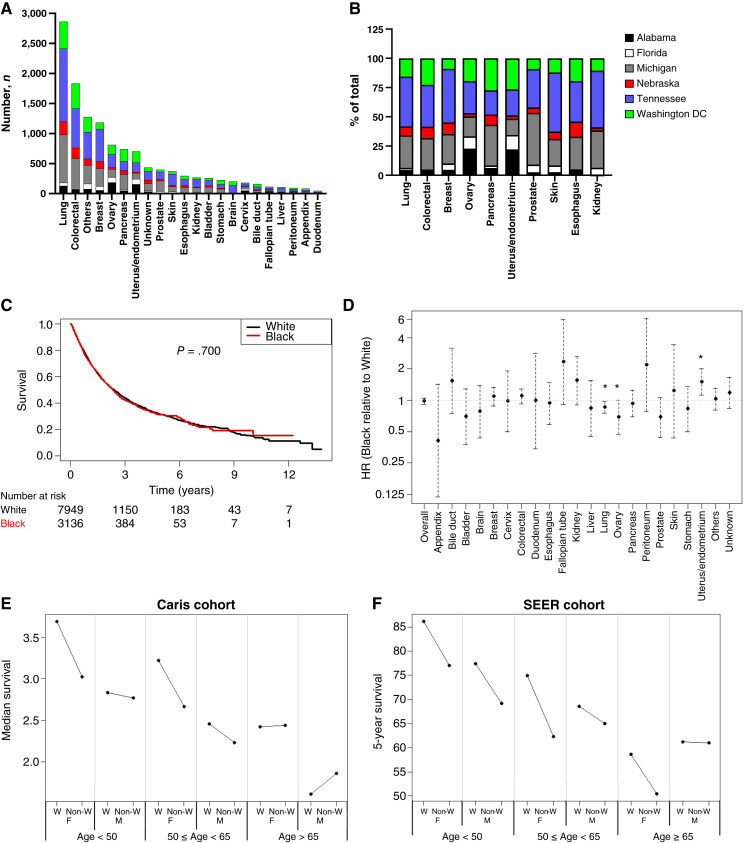
Tumor lineages of the cohort and analysis of OS by race. **A,** Total numbers of each tumor type broken down by study site. **B,** Percentage of the total made up by each study site for the top 10 most represented tumor types. **C,** Kaplan–Meier curve for OS of White patients (black line) and Black patients (red line) among molecularly-tested cohort. **D,** COXPH analyzing the effect of race on OS in individual tumor types. *, *P* < 0.05. **E** and **F,** Median survival among the Caris cohort (**E**) or 5-year survival among the SEER cohort (**F**) was plotted for male and female White and non-White patients in three different age groups (<50 years; 50 ≤ years < 65; and ≥65).

A total of 12,627 patients whose tumors underwent molecular testing at Caris Life Sciences between the years of 2010 to 2020 were included from six sites throughout the United States, with the largest fractions coming from Tennessee and Michigan, followed by Washington, DC (Table 1). Sex (assigned at birth) distribution was 58% male and 42% female. The median age was 62.3 years, with Nebraska and Tennessee having a significantly higher median age of 64.4 and 63.0 years, respectively. Self-reported racial groupings included White (63%), Black (25%), and “other” (12%), which comprised racial minorities underpowered for most analyses (e.g., Asian and American Indian), and patients with unknown race. Other demographic variables analyzed were poverty level (defined as the percentage of people in the patient’s zip code who are living below the federal poverty level), and location [defined by rural–urban commuting area (RUCA) codes]. Lower RUCA codes indicate urban areas and higher codes indicate rural areas (Supplementary Table S1). Clinical staging information was only available for 5,267 patients—a limitation of data extraction from medical records/insurance claims data (20–23)—but indicated that a majority of tumors (76%) were stage III and IV, which is in keeping with the typical use of molecular testing for advanced disease. Twenty tumor lineages were captured among the patients, with lung, colorectal, breast, ovary, pancreas, endometrial, prostate, and skin being the most common (Fig. 1A and B). The comparison of our data to established cancer prevalence data showed that the observed frequency of cancer cases per population matched very closely with the expected frequency (Supplementary Fig. S1), confirming that our cohort reliably captured the true prevalence of cancer among the population.

**Table 1:** Cohort demographics

	Overall	AL	FL	MI	NE	TN	DC	*P* value	Number, *N*
Age (range)	62.276 (55.00–71.00)	62.568 (56.00–71.00)	61.250 (54.00–70.00)	61.355 (54.00–70.00)	64.424 (58.00–73.00)	62.957 (56.00–72.00)	61.457 (54.00–70.00)	***<0.001***	12,627
Poverty % (range)	14.880 (6.60–20.50)	17.730 (11.20–22.70)	14.835 (9.30–19.67)	14.971 (6.25–19.00)	8.887 (4.60–12.10)	18.331 (7.40–26.20)	9.224 (5.00–12.10)	***<0.001***	12,371
Sex, no. (%)								***<0.001***	12,624
Male	7,289 (58%)	706 (75%)	403 (73%)	1,727 (54%)	518 (55%)	2,542 (55%)	1,393 (59%)		
Female	5,335 (42%)	234 (25%)	149 (27%)	1,498 (46%)	428 (45%)	2,077 (45%)	949 (41%)		
Race, no. (%)								***<0.001***	12,627
White	7,949 (63%)	628 (67%)	413 (75%)	2,080 (64%)	640 (68%)	2,871 (62%)	1,317 (56%)		
Black	3,136 (25%)	257 (27%)	91 (16%)	689 (21%)	19 (2%)	1,479 (32%)	601 (26%)		
Other	1,542 (12%)	55 (6%)	49 (9%)	456 (14%)	287 (30%)	269 (6%)	426 (18%)		
RUCA code, No. (%)^a^								***<0.001***	12,350
1	9,250 (75%)	644 (70%)	525 (98%)	2,428 (78%)	686 (74%)	2,897 (63%)	2,070 (90%)		
2–3	1,194 (10%)	126 (14%)	2 (0%)	232 (8%)	77 (8%)	557 (12%)	200 (9%)		
4–6	714 (6%)	52 (6%)	8 (1%)	102 (3%)	77 (8%)	459 (10%)	16 (1%)		
7–10	1,192 (10%)	96 (10%)	1 (0%)	331 (11%)	91 (10%)	658 (14%)	15 (1%)		
Stage, no. (%)								***<0.001***	5,267
I	583 (11%)	37 (18%)	19 (16%)	63 (11%)	26 (8%)	365 (11%)	73 (9%)		
II	691 (13%)	28 (14%)	21 (18%)	83 (14%)	48 (15%)	386 (12%)	125 (15%)		
III	1,352 (26%)	76 (37%)	30 (26%)	171 (29%)	69 (21%)	811 (25%)	195 (23%)		
IV	2,641 (50%)	63 (31%)	47 (40%)	271 (46%)	183 (56%)	1,636 (51%)	441 (53%)		

The total cohort (*n* = 12,627) was analyzed by *χ*^2^ test for significant differences in the distribution of sex, race, RUCA code, and stage between the sites.

Significant *P* values are bolded and italicized.

a RUCA, rural–urban commuting area. See Supplementary Table S1 for RUCA descriptions.

### Racial disparities among patients with molecularly-tested tumors

Kaplan–Meier analysis demonstrated that when patients underwent molecular testing for their cancer, there was no difference in OS between White and Black patients with all tumor types combined (*P* = 0.700; Fig. 1C), contrasting with well-documented racial disparities among the general cancer population (2, 4). Small but significant improvements in OS were observed for Black patients with lung and ovarian cancers compared with White patients, whereas the opposite was observed for Black patients with endometrial cancer (Fig. 1D). These data align with the increased incidence of uterine serous carcinoma—a more aggressive subtype with worse prognosis—among Black women (24). Although staging information was less frequently available, longer OS was commensurate with earlier stage, as expected (*P* < 0.001), and Black patients were slightly more likely than White patients to have later-stage disease (47.9% *vs.* 53.3% stage IV; *P* < 0.001; Supplementary Fig. S2).

Because individual demographic characteristics may not fully reveal the impact of the intersection of multiple factors on health outcomes (2), we analyzed the intersection of age, race, and sex in our cohort. Our data show that only younger non-White patients experienced a shorter median survival compared with White patients, particularly when considering female patients (Fig. 1E). Because direct comparison to a comparable nonmolecularly-tested cohort is currently not feasible, we compared these trends to the general population of cancer patients using 5-year survival data from the Surveillance, Epidemiology, and End Results (SEER) program (25). Although the same trends were observed, the racial disparities were more striking and persisted in all ages of both sexes (Fig. 1F). Because cancer registries including SEER report greater mortality of Black men compared with White men (26), we also examined the mortality of Black men in our cohort by examining 3-year survival rates. Interestingly, we found that younger Black men also had worse 3-year survival rates compared with White men (<50: 38.0% *vs.* 46.5% and 50–64: 34.4% *vs.* 43.1%), but this was reversed at older ages (≥65: 38.1% *vs.* 31.0%). Thus, our cohort does reflect the higher mortality of Black men that is observed in cancer registries but demonstrates further nuances depending on age.

### Factors affecting overall survival

Using Cox proportional hazards (COXPH) regression, we delved further into factors affecting patient outcomes. Here, we observed that White race was an independent predictor of longer OS, suggesting context-dependent racial disparities may exist that were not captured in the Kaplan–Meier analysis (Table 2). When analyzing other demographic features, older patients of all races, as well as White males but not White females, experienced shorter OS. Rural zip code was also significantly associated with worse OS, but this effect was driven solely by the Michigan site (Table 2) and could be related to poverty levels among higher RUCA codes in Michigan (Supplementary Fig. S3A and S3B). There were no differences in survival among higher RUCA codes 7–10 (*P* = 0.835; Supplementary Fig. S3C). These data suggest that location or poverty level were not highly important predictors of outcome for patients undergoing molecular testing at most of the study sites.

**Table 2:** COXPH model for all patients in the cohort

	Overall		AL		FL		MI		NE		TN		DC	
	HR	*P* value	HR	*P* value	HR	*P* value	HR	*P* value	HR	*P* value	HR	*P* value	HR	*P* value
Rural zip code (RUCA 7–10)^a^	1.13	***0.012***	0.80	0.311	NA	NA	1.63	***<0.001***	1.05	0.783	1.04	0.470	0.63	0.427
White race	0.60	***0.004***	0.28	0.074	2.81	0.361	0.32	***0.012***	0.92	0.900	0.62	***0.049***	0.81	0.641
Age (per 10 years) for non-White race	1.05	***0.037***	1.05	0.608	1.05	0.786	0.94	0.306	1.15	0.119	1.08	***0.013***	1.01	0.926
Age (per 10 years) for White race	1.12	***<0.001***	1.24	***0.002***	0.86	0.107	1.16	***<0.001***	1.15	***0.010***	1.13	***<0.001***	1.04	0.435
Male sex for the non-White race	1.06	***0.265***	0.96	0.874	1.90	0.246	1.17	0.300	1.11	0.587	1.10	0.188	0.85	0.246
Male sex for the White race	1.23	***<0.001***	1.77	***0.009***	0.85	0.689	1.09	0.373	1.04	0.726	1.32	***<0.001***	1.21	0.093
*APC* mut	0.79	***<0.001***	0.83	0.536	0.87	0.821	0.86	0.394	0.73	0.112	0.81	***0.027***	0.69	***0.036***
*TP53* mut	1.53	***<0.001***	1.39	***0.019***	1.54	***0.040***	1.56	***<0.001***	1.52	***<0.001***	1.59	***<0.001***	1.39	***<0.001***
*EGFR*-mut	0.69	***0.001***	0.16	0.070	2.77	0.220	0.78	0.338	0.51	0.093	0.64	***0.007***	0.99	0.963
*STK11* mut	1.31	***0.002***	2.60	***0.001***	2.45	0.286	1.50	***0.027***	1.20	0.463	1.24	0.084	1.00	0.986
*KRAS* mut	1.14	***0.002***	1.50	***0.022***	0.90	0.773	1.24	***0.034***	1.41	***0.010***	1.05	0.455	1.08	0.489

COXPH analysis incorporating all variables for patients, stratified by site.

Significant *P* values are bolded and italicized.

a RUCA, rural–urban commuting area. See Supplementary Table S1 for RUCA descriptions.

Examination of gene mutations revealed that *APC* and *EGFR* mutations were associated with better OS in the entire cohort (Table 2) and in their hallmark tumor types [colorectal cancer and non-small cell lung cancer (NSCLC), respectively; Supplementary Fig. S4A and S4B]. Conversely, *TP53*, *STK11*, and *KRAS* mutations were associated with worse OS in the entire cohort (Table 2); *STK11* and *KRAS* mutations were furthermore associated with worse OS in their hallmark tumor types (NSCLC and pancreatic/colorectal, respectively; Supplementary Fig. S4C–S4E). The difference in OS based on *TP53* mutation status was particularly striking in breast, prostate, and uterine cancers and less striking in NSCLC, colon, ovarian, and pancreatic cancers (Supplementary Fig. S4F–S4L). Additional analyses confirmed that our findings held true in patients with at least 6 months follow-up and confirmed the association of higher stage with worse OS, as expected (Supplementary Tables S2 and S3).

### Genomic characteristics of the cohort

**Figure 2:**
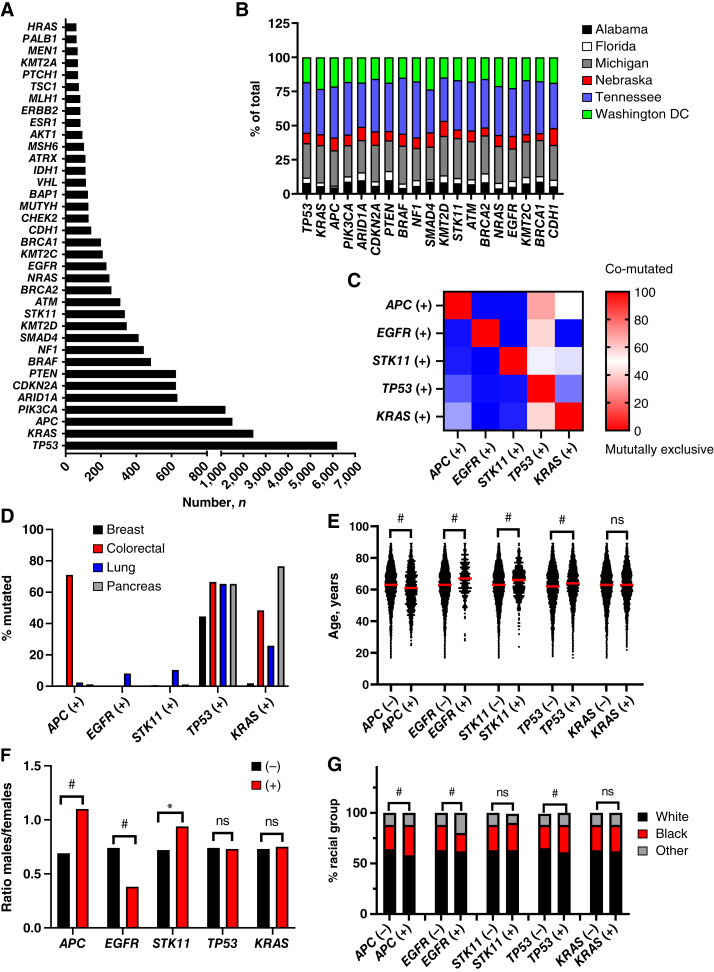
Genomic features and clinico-demographic associations of cohort. **A,** Total number of tumors positive for each mutated gene. **B,** Percentage of total tumors from each study site for the top 20 most commonly mutated genes. **C,** Prevalence of co-mutations among *APC*, *EGFR*, *STK11*, *TP53*, and *KRAS*. Red indicates strong co-mutation whereas blue indicates mutual exclusivity. **D,** Percentage of indicated tumor types with mutations in *APC*, *EGFR*, *STK11*, *TP53*, and *KRAS*. **E,** Age of patients among (−) and (+) gene mutation cohorts. The red line indicates the median age. **F,** Ratio of males to females in (−) and (+) gene mutation cohorts. **G,** Percentage of total (−) and (+) cohorts composed by indicated racial groups. *, *P* < 0.05; **, *P* < 0.01; #, *P* < 0.001; ns, not significant.

**Figure 3:**
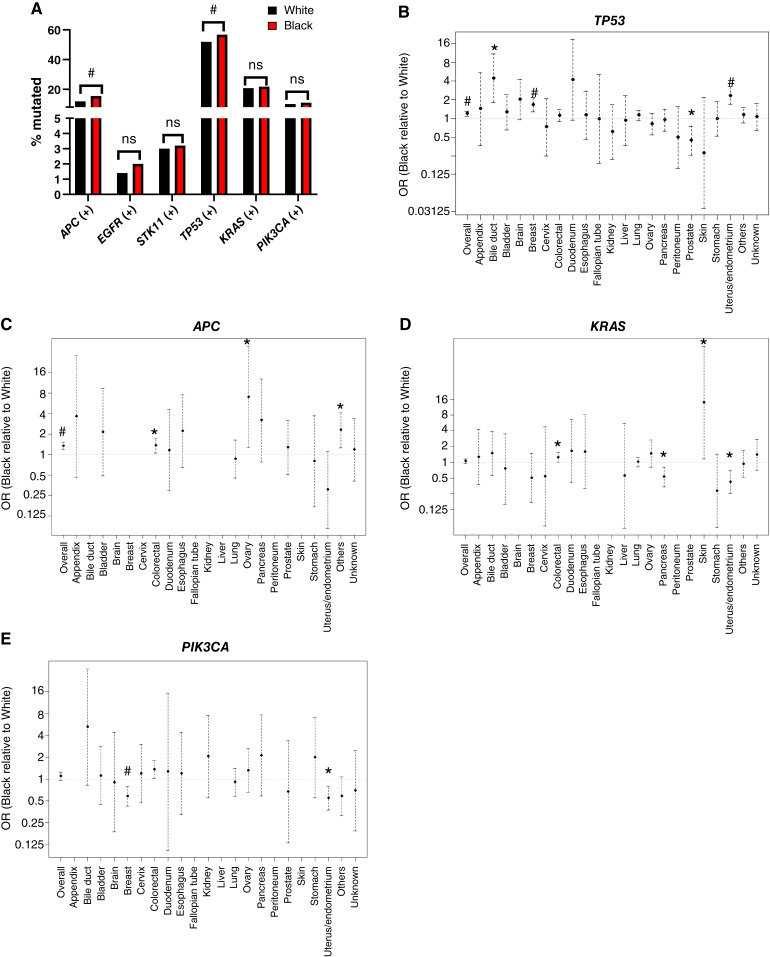
Gene mutations by race. **A,** Percentage of tumors from Black *versus* White patients that are positive for the indicated gene mutations. #, *P* < 0.001; ns, not significant. **B–E,** Probability of Black patients having a *TP53* (**B**), *APC* (**C**), *KRAS* (**D**), or *PIK3CA* (**E**) mutation relative to White patients for each individual tumor type. *, *P* < 0.05; #, *P* < 0.001. When data are not shown, we did not produce confidence intervals because of low gene mutation prevalence for the indicated cancer type.

We characterized the genomic characteristics of the cohort by examining genes with a pathogenic or likely pathogenic mutation in at least 0.5% of the cohort. In order to ensure our results were generalizable, we also excluded genes that were not analyzed in at least 25% of the cohort (Fig. 2A and B). Copy number and fusion data were not analyzed because they were infrequent and not consistently reported across sites (Supplementary Fig. S5). *TP53* mutations were the most common (53% prevalence overall), followed by *KRAS* (21%), *APC* (13%), and *PIK3CA* (10%; Fig. 2A). *TP53* mutations were less frequent in breast compared with colorectal, lung, and pancreatic cancers (Fig. 2C; Supplementary Fig. S6). Analyzing the five genes that were identified as affecting survival outcomes (Table 2), we observed that these genes—with the exception of *TP53*—were mostly mutually exclusive and enriched in their hallmark tumor types (Fig. 2C and D) as reported in the literature (i.e., *APC* mutations in colorectal cancer; *KRAS* mutations in colorectal, lung, and pancreas; and *EGFR* and *STK11* mutations in lung cancer; refs. 27–29). These mutations were also associated with patient demographics including age (*EGFR*, *STK11*, and *TP53* with older age, and *APC* with younger age), sex (*APC* and *STK11* with male sex and *EGFR* with female sex), and poverty levels (*EGFR* with lower poverty, but not geographic location; Fig. 2E and F; Supplementary Fig. S7A and S7B). Black patients were underrepresented among *EGFR*-mutated cancers but were slightly overrepresented among *APC*- or *TP53*-mutated cancers (Fig. 2G). Moreover, the overall prevalences of *APC* and *TP53* mutations were significantly greater among Black than White patients, agreeing with a prior pan-cancer racial analysis of The Cancer Genome Atlas (30). However, these differences were small, and there were no significant differences in the overall percentages of *EGFR*, *STK11*, or *KRAS* between White and Black patients (Fig. 3A).

Because a pooled pan-cancer analysis of genomic differences between races is likely to be diluted by the vast heterogeneity of tumor types, we next investigated genomic differences at a tumor lineage level. This analysis revealed racial differences in alteration frequency of the most common pan-cancer gene alterations (*TP53*, *APC*, *KRAS*, and *PIK3CA*). Black patients had a greater risk of *TP53* mutation in bile duct, breast, and endometrial cancers, *APC* mutations in colorectal and ovarian cancers, and *KRAS* in colon and skin cancers (Fig. 3B–D). Conversely, Black patients had a lesser risk of *TP53* mutations in prostate cancer, *KRAS* mutations in pancreatic and endometrial cancers, and *PIK3CA* mutations in breast and endometrial cancers (Fig. 3B, D, and E). Together, these data illustrate the potential for racial disparities to be driven by genomic differences in specific tumor types and further emphasize the need for improved access to molecular testing for racial minorities.

### Comparison of treatment-associated outcomes and effect of mutation status

**Figure 4:**
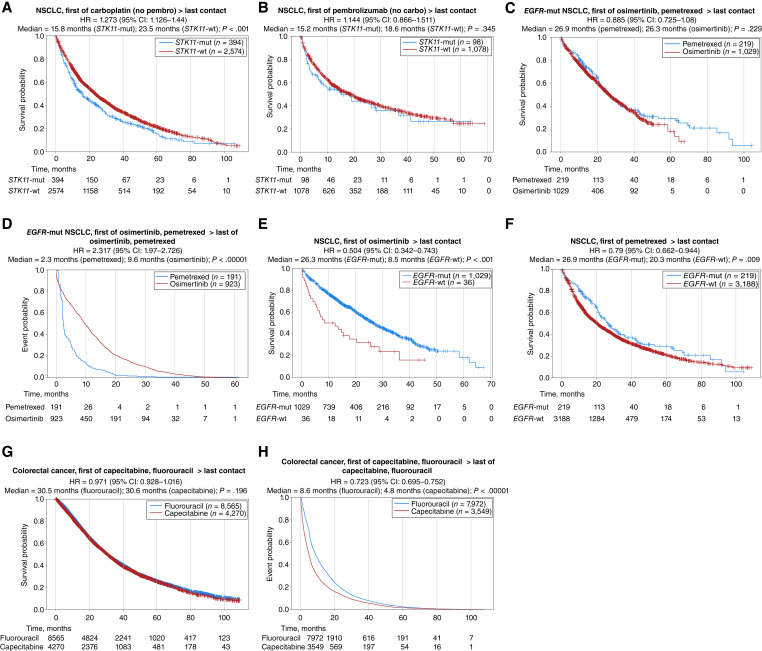
Analysis of treatment-associated outcomes and effect of mutation status among patients in Caris CODEai clinico-genomic database. **A** and **B,** Kaplan–Meier analysis comparing posttreatment OS between patients with *STK11*-mutated (mut, blue lines) and *STK11*-wild type (wt, red lines) NSCLC treated with (**A**) carboplatin (no pembrolizumab) and (**B**) pembrolizumab (no carboplatin). **C** and **D,** Kaplan–Meier analysis comparing (**C**) posttreatment OS and (**D**) TOT between patients treated with pemetrexed (blue lines) and osimertinib (red lines) for *EGFR*-mut NSCLC. **E** and **F,** Kaplan–Meier analysis comparing posttreatment OS between patients with *EGFR*-mut (blue lines) and *EGFR*-wt (red lines) NSCLC treated with (**E**) osimertinib or (**F**) pemetrexed. **G** and **H,** Kaplan–Meier analysis comparing (**G**) posttreatment OS and (**H**) TOT between patients treated with fluorouracil (blue lines) and capecitabine (red lines) for colorectal cancer.

Next, we analyzed therapeutic responses in groups defined by mutation status—focusing on the top five mutations we found to be associated with OS, as described above (*TP53*, *STK11*, *EGFR*, *APC*, and *KRAS*). *STK11* mutations conferred better survival among patients treated with carboplatin, but not pembrolizumab (Fig. 4A and B). Interestingly, patients with *EGFR*-mutated NSCLC experienced similar outcomes following treatment with the EGFR inhibitor osimertinib compared with pemetrexed chemotherapy; however, total TOT was significantly longer with osimertinib compared with pemetrexed (Fig. 4C and D). Likewise, *EGFR* mutations conferred a dramatic posttreatment OS benefit for osimertinib but less so for pemetrexed, illustrating the benefit of molecularly selected EGFR-targeted therapy (Fig. 4E and F). Among all patients with colorectal cancer, fluorouracil yielded similar posttreatment OS as capecitabine but longer TOT (Fig. 4G and H), with no effect of either *APC* or *KRAS* mutation status. Expectedly, patients with *TP53*-mutated tumors (all tumor types combined) who were treated with carboplatin, paclitaxel, or fluorouracil chemotherapy experienced slightly longer OS than patients with *TP53*-wt tumors (Supplementary Fig. S8).

## Discussion

The lack of diversity in genomic datasets has hampered research on health disparities (1). This multiinstitutional cohort of more than 12,000 patients who underwent genomic testing in the United States is the largest to date that includes a significant proportion of Black patients and provides new insights into factors driving racial disparities. Strikingly, our results imply that molecularly tested patients have similar outcomes based on race, although some disparities persist at a more granular level (i.e., in specific cancers and younger ages). Some of these more granular differences may be driven by ancestry-associated features for specific cancers, such as the increased prevalence of *TP53*-mut uterine serous carcinomas in Black women (24). The leveling out of racial disparities at older ages could be due to generally worse outcomes among this demographic (31, 32), which could override racial differences in outcomes. As we observed for racial health disparities, disparities stemming from geographic location and poverty level also seem minimal when patients have access to molecular testing. Future research should aim to better elucidate the reasons for persistent disparities that exist despite the implementation of molecular testing. For instance, a prospective cohort study showed that Black patients with endometrial cancer were less frequently initiated on targeted therapy or enrolled in clinical trials compared with non-Black patients, despite undergoing molecular testing for their tumor (33).

Among all the genomic alterations examined in this study, only mutations in five genes—*APC*, *EGFR*, *STK11*, *TP53*, and *KRAS*—were independent predictors of OS at a pan-cancer level. The two genes with the most consistent associations with outcomes among all study sites, *TP53* and *KRAS*, have previously been associated with shorter survival in cancer (34). Interestingly, although *EGFR* mutations were only significantly associated with longer survival at the Tennessee site, *EGFR* mutations were significantly associated with lower poverty levels. These results can possibly be explained by the established correlation between smoking and lower *EGFR* mutation rates (35), lower socioeconomic class (36), and worse survival (35). Thus, genomic differences may be related to health disparities due to other confounding demographic variables.

Prior studies have indicated that African American ancestry is associated with the enrichment of specific gene mutations including *TP53* and *FBXW7*, and European American ancestry with *PIK3CA* mutations; however, most associations were only apparent within specific cancer lineages due to the varying prevalence of cancer subtypes across ancestries (5, 30). Although we did not specifically examine ancestry, we similarly observed racial differences in *TP53*, *APC*, *KRAS*, and *PIK3CA* mutation frequency in select tumor types. Taken together, our data highlights that genomic differences may not be major drivers of racial disparities at a pan-cancer level, but they must be considered in specific tumor types.

Finally, the analysis of survival outcomes in this large, multiinstitutional cohort adds further real-world context to some previously reported therapeutic response trends. In colorectal cancer, our data confirms prior studies demonstrating the relative equivalency of chemotherapy regimens including fluorouracil or its prodrug capecitabine (37). In *EGFR*-mutated NSCLC, we observed longer TOT with osimertinib compared with pemetrexed with no difference in posttreatment OS, mirroring results of the phase 3 AURA3 trial that demonstrated longer PFS in osimertinib-treated patients versus platinum/pemetrexed-treated patients, but no meaningful prolongation of OS (38, 39). Regarding *STK11* mutation status, there are conflicting studies on the role of these mutations in promoting pembrolizumab resistance in NSCLC (40–42). Our results, which indicate that *STK11* mutations confer worse OS following carboplatin but not pembrolizumab treatment, seem in agreement with studies finding no effect of *STK11* mutations on pembrolizumab response in clinical trial cohorts (43, 44). Together with a case report of an individual with *STK11*/*KRAS* co-mutated advanced lung cancer with near complete response to pembrolizumab and platinum-doublet therapy (45), these data suggest that *STK11* mutation should not be used as a strict exclusion criterion for immune checkpoint blockade in NSCLC. One limitation is that our analysis of OS must be interpreted with caution because of our inability to control for variables associated with treatment decisions, such as performance status, or prognostic variables, such as stage and grade. The treatment-defined cohorts are also exclusive of patients receiving the comparator therapy, which may exclude some real-world treatment scenarios.

An additional limitation of this study was our inability to analyze other minoritized groups because of low representation or missing racial information. Nonetheless, our approach allowed a comparison of the two predominant racial groups in the United States, which are frequently compared in health disparity studies (4). Although we cannot comment on the exact cause of reduced disparities in this cohort—or eliminate some inherent and unavoidable biases of this retrospective study—we speculate that with greater use of testing, there is a commensurate increase in biomarker-directed cancer care, which is known to improve outcomes for patients with cancer (46–48). The benefit of more widespread implementation of molecular testing is reflected in recent recommendations from the National Comprehensive Cancer Network (NCCN) expanding the criteria for broad-based molecular testing for several cancer types (49–51). However, systemic barriers including physician and patient knowledge of precision cancer medicine and appropriate insurance coverage can limit access to testing (52). Patients with insurance access are more likely to undergo genetic testing, and the type of insurance can dictate the extent of coverage (53–55). Therefore, potential confounders in our dataset include a lack of information on insurance access or type of insurance. We also acknowledge a potential cohort bias because our analysis included academic medical centers as opposed to community hospitals, which are known to impact patient care and outcomes, regardless of demographic or socioeconomic factors (56–59). Demographic and socioeconomic factors can, however, impact where patients receive care, but there is conflicting evidence on the propensity for non-Whites, uninsured, and Medicaid patients to receive care at academic versus community hospitals (59–61). Thus, further research will be required to extrapolate our results to the community hospital setting and to fully understand the effect of insurance status on molecular testing and outcomes with regard to race. Finally, because of a lack of a true “control” group of patients who did not receive molecular testing, we instead compared our findings to those of the general population of patients with cancer represented in the SEER database, which displayed the expected disparities that have been well-documented in numerous studies. Furthermore, to confirm that our cohort was representative of cancer incidence in the general population, we verified that expected cancer counts per population in our dataset were comparable with the Puckrein dataset produced by the National Minority Quality Forum (Supplementary Fig. S1). Therefore, we conclude that the comparison of our findings to the general cancer population is a valid approach to such disparities research, although our findings will require verification in additional cohorts.

This study suggests the important role that access to molecular testing might play in improving health equity for oncology patients, and represents a stepping stone to further research into this challenging but important topic. In addition to addressing known systemic barriers, access to testing may be further enhanced by the development of liquid biopsy, which is less invasive and more cost-effective, overcomes issues with limited tissue availability, and can be performed serially throughout the course of treatment in flexible settings (62–64). Therefore, with continued improvements in technology, education, and implementation, the lack of access to molecular testing can potentially be reduced, possibly contributing to increased health equity for oncology patients.

## Supplementary Material

Supplementary Figure S1 Comparison of Caris cohort data with Puckrein dataset

Supplementary Figure S2 Overall survival of cohort by stage

Supplementary Figure S3 Overall survival by RUCA code and poverty

Supplementary Fiugre S4 Overall survival by RUCA code and poverty

Supplementary Figure S5 Copy number alterations (CNAs) and fusions among cohort

Supplementary Figure S6 Prevalence of common mutations in tumor subtypes

Supplementary Figure S7 Gene mutations according to poverty level and RUCA codes

Supplementary Figure S8 Comparison of overall survival among chemotherapy-treated TP53-mutated and TP53-wild type patients in Caris CODEai clinico-genomic database

Supplementary Table S1 Description of rural-urban commuting area (RUCA) codes

Supplementary Table S2 COXPH model for patients with at least 6-mo overall survival

Supplementary Table S3 COXPH model for patients with stage information
