# Supplementary material for: Health Disparities among Patients with Cancer Who Received Molecular Testing for Biomarker-Directed Therapy
Source: Cancer Res Commun. 2024 Oct 4;4(10):2598–609. doi: 10.1158/2767-9764.CRC-24-0321 (PMC11450693; doi:10.1158/2767-9764.CRC-24-0321)
Supplement: Supplementary Figure S1 — Comparison of Caris cohort data with Puckrein dataset [file crc-24-0321_supplementary_figure_s1_suppsf1.docx]

**Supplementary Figure S1. Comparison of Caris cohort data with Puckrein dataset.** Lung **(A)** and colon **(B)** cancer counts per population by 3-digit zip code for the year 2019. Black line, Puckrein data; red line, Caris data; blue line, expected cancer counts per population for Caris data based on Puckrein data. The Puckrein dataset is the result of the “Zip Code Analysis Project” spearheaded by the National Minority Quality Forum, linking demographic and clinical data to zip codes in order to advance health disparities research. Due to availability of incidence year for Caris data, only Tennessee, Michigan, and Washington DC sites were included in the comparison (n = 10,188).

**
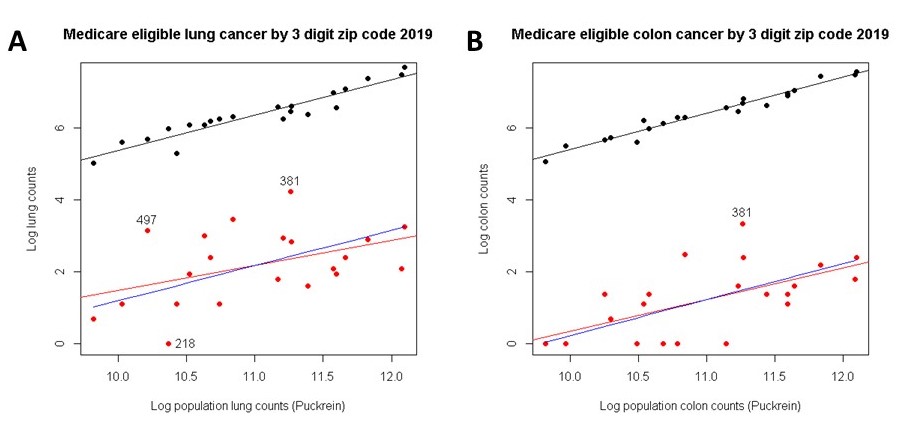
**
