# Supplementary material for: Health Disparities among Patients with Cancer Who Received Molecular Testing for Biomarker-Directed Therapy
Source: Cancer Res Commun. 2024 Oct 4;4(10):2598–609. doi: 10.1158/2767-9764.CRC-24-0321 (PMC11450693; doi:10.1158/2767-9764.CRC-24-0321)

**Supplementary Figure S2. Overall survival of cohort by stage.** **(A)** Kaplan-Meier survival curve for all patients with available stage data, stratified by stage (I, II, II, IV). **(B)** Percentage of White and Black patients with stage I-IV disease. ***P* < 0.001.


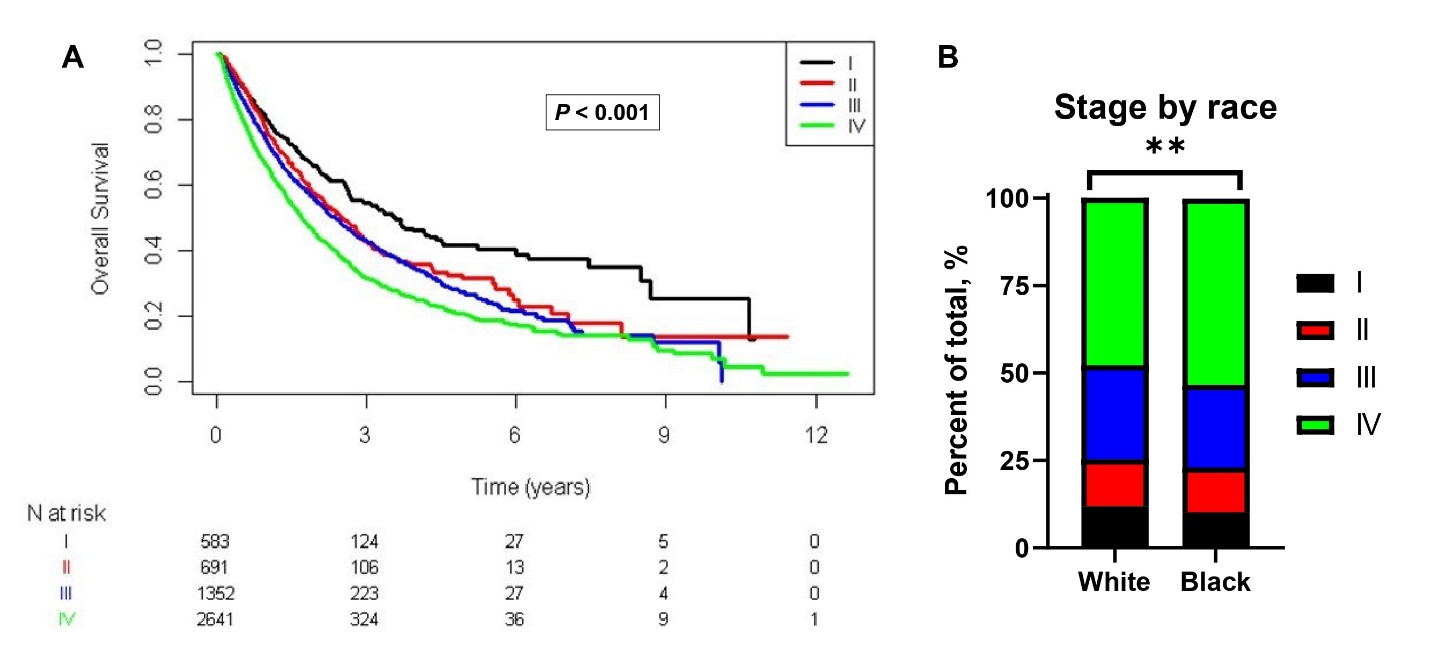

Supplement: Supplementary Figure S2 — Overall survival of cohort by stage [file crc-24-0321_supplementary_figure_s2_suppsf2.docx]
