# Supplementary material for: Health Disparities among Patients with Cancer Who Received Molecular Testing for Biomarker-Directed Therapy
Source: Cancer Res Commun. 2024 Oct 4;4(10):2598–609. doi: 10.1158/2767-9764.CRC-24-0321 (PMC11450693; doi:10.1158/2767-9764.CRC-24-0321)
Supplement: Supplementary Figure S3 — Overall survival by RUCA code and poverty [file crc-24-0321_supplementary_figure_s3_suppsf3.docx]

**Supplementary Figure S3. Overall survival by RUCA code and poverty. (A)** Kaplan-Meier curves describing overall survival defined by RUCA codes and poverty at the Michigan site. **(B)** Kaplan-Meier curves describing overall survival defined by RUCA codes and poverty at all other sites (excluding Michigan). **(C)** Kaplan-Meier curves describing overall survival among higher RUCA codes (7-10).

**
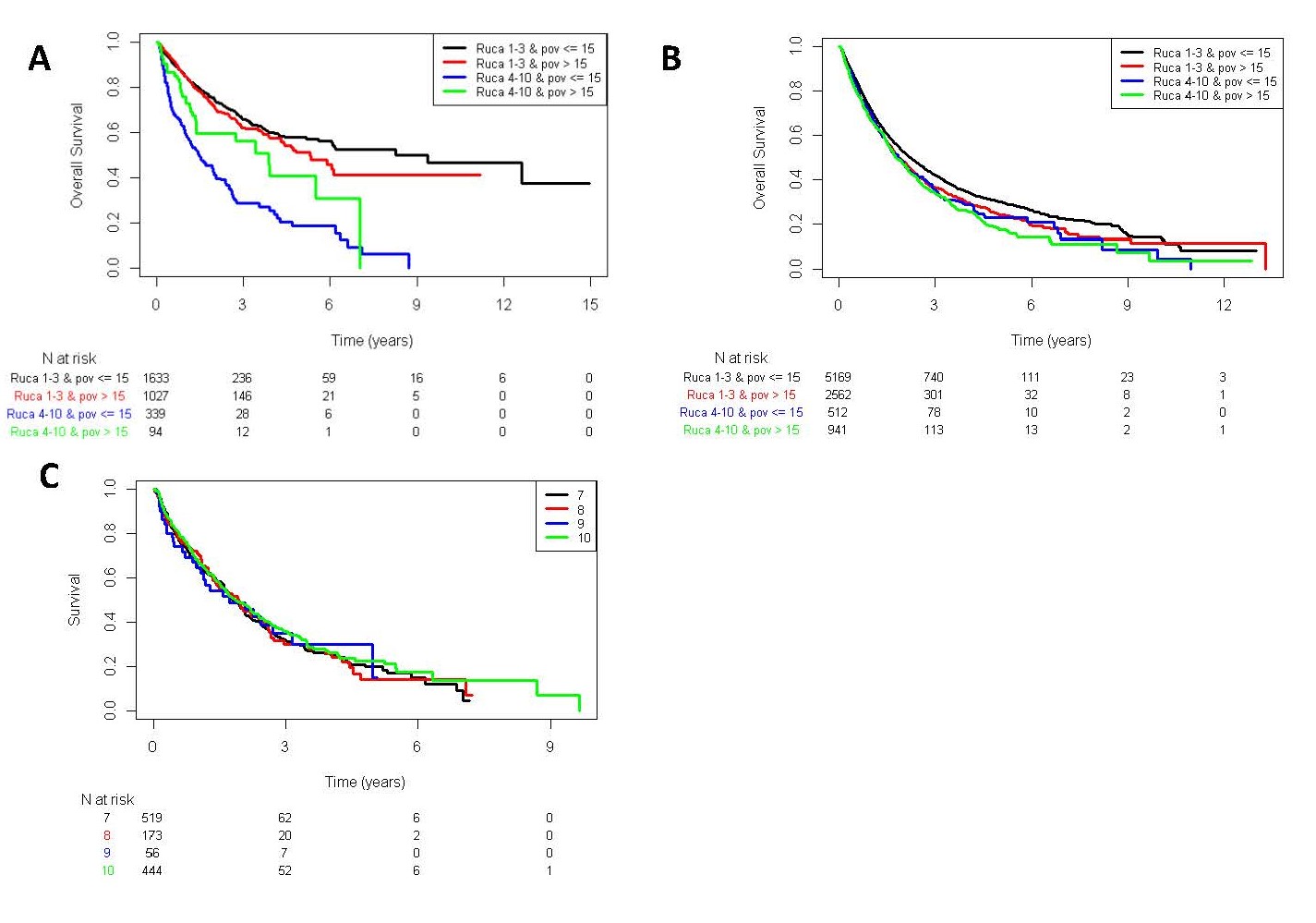
**

*P* < .001

*P* < .001

*P* = .835
