# Supplementary material for: Health Disparities among Patients with Cancer Who Received Molecular Testing for Biomarker-Directed Therapy
Source: Cancer Res Commun. 2024 Oct 4;4(10):2598–609. doi: 10.1158/2767-9764.CRC-24-0321 (PMC11450693; doi:10.1158/2767-9764.CRC-24-0321)
Supplement: Supplementary Fiugre S4 — Overall survival by RUCA code and poverty [file crc-24-0321_supplementary_fiugre_s4_suppsf4.docx]

**Supplementary Figure S4. Overall survival of mutation-defined cohorts among hallmark tumor types.** Kaplan-Meier curves describing overall survival (OS) of colorectal cancer *APC*-mutated versus *APC*-wild type patients **(A)**, non-small cell lung cancer (NSCLC) *EGFR*-mutated versus *EGFR*-wild type patients **(B)**, NSCLC *STK11*-mutated versus *STK11*-wild type patients **(C)**, pancreatic cancer *KRAS*-mutated versus *KRAS*-wild type **(D)**, colorectal cancer *KRAS*-mutated versus *KRAS*-wild type **(E)**, breast cancer *TP53*-mutated versus *TP53*-wild type **(F)**, prostate cancer *TP53*-mutated versus *TP53*-wild type **(G)**, uterine cancer *TP53*-mutated versus *TP53*-wild type **(H)**, NSCLC *TP53*-mutated versus *TP53*-wild type **(I)**, colorectal cancer *TP53*-mutated versus *TP53*-wild type **(J)**, ovarian cancer *TP53*-mutated versus *TP53*-wild type **(K)**, and pancreatic cancer *TP53*-mutated versus *TP53*-wild type **(L)**. Mut = mutated (blue); wt = wild type (red). OS shown from tissue collection to last contact, based on insurance claims data.

**
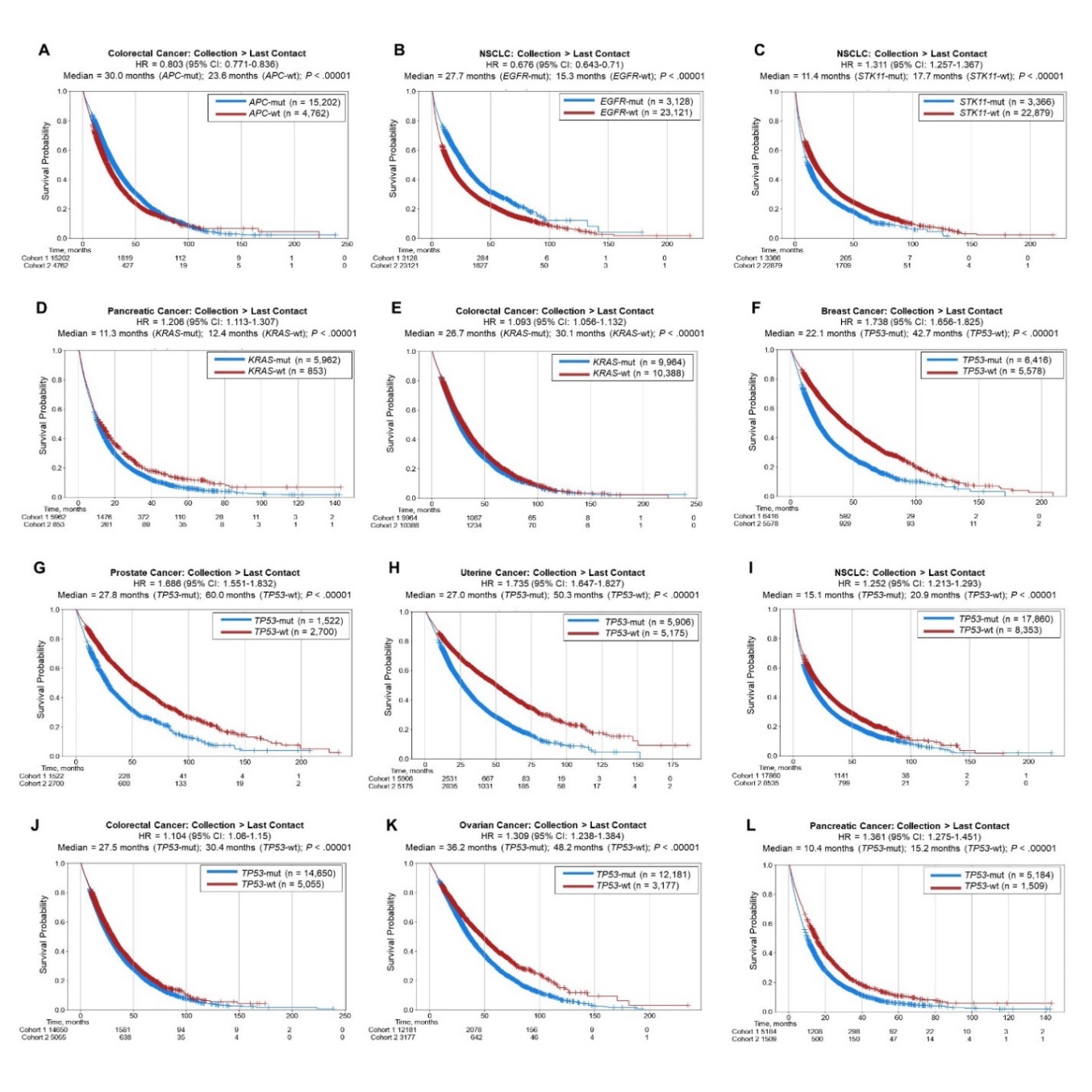
**
