# Supplementary material for: Health Disparities among Patients with Cancer Who Received Molecular Testing for Biomarker-Directed Therapy
Source: Cancer Res Commun. 2024 Oct 4;4(10):2598–609. doi: 10.1158/2767-9764.CRC-24-0321 (PMC11450693; doi:10.1158/2767-9764.CRC-24-0321)
Supplement: Supplementary Figure S5 — Copy number alterations (CNAs) and fusions among cohort [file crc-24-0321_supplementary_figure_s5_suppsf5.docx]

**Supplementary Figure S5. Copy number alterations (CNAs) and fusions among cohort.** CNAs and fusions were identified in tumors from the Michigan, Nebraska, Tennessee, and Washington DC sites. The most prevalent alterations are shown (less than 200 tumors displayed each of these alterations among the four sites tested).

**
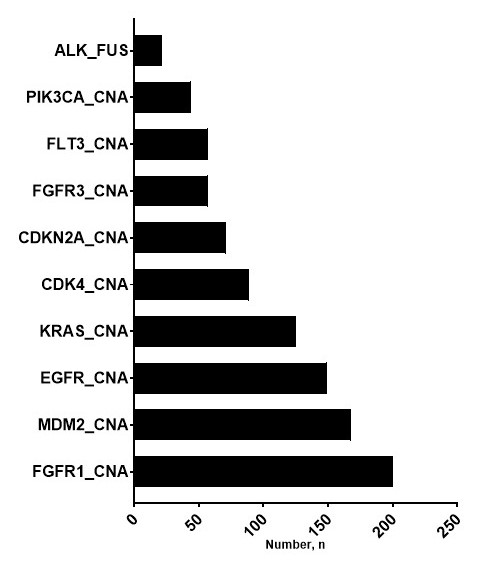
**
