# Supplementary material for: Health Disparities among Patients with Cancer Who Received Molecular Testing for Biomarker-Directed Therapy
Source: Cancer Res Commun. 2024 Oct 4;4(10):2598–609. doi: 10.1158/2767-9764.CRC-24-0321 (PMC11450693; doi:10.1158/2767-9764.CRC-24-0321)
Supplement: Supplementary Figure S6 — Prevalence of common mutations in tumor subtypes [file crc-24-0321_supplementary_figure_s6_suppsf6.docx]

**Supplementary Figure S6.** **Prevalence of common mutations in tumor subtypes.** Percentage of breast **(A)**, colorectal **(B)**, lung **(C)**, and pancreatic **(D)** tumors displaying *APC*, *EGFR*, *STK11*, *TP53*, and *APC* mutations. *P* < .001 for all (-) vs (+) comparisons.

**
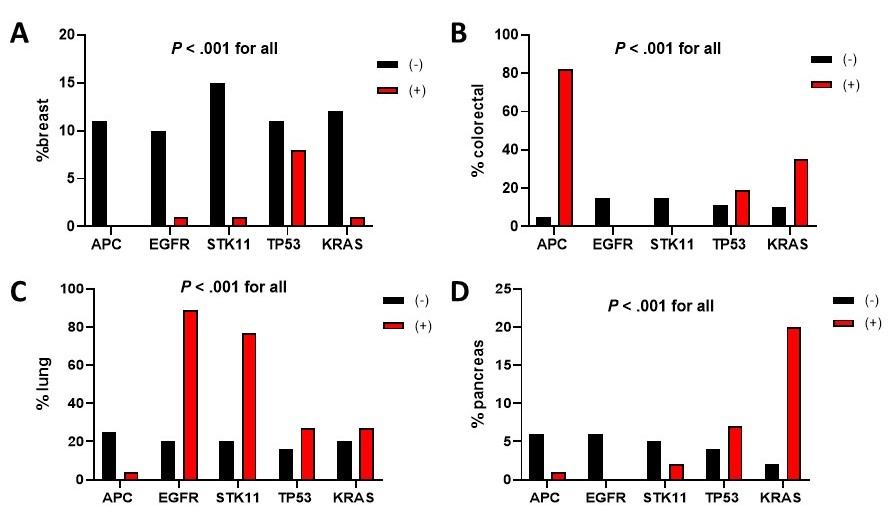
**
