# Supplementary material for: Health Disparities among Patients with Cancer Who Received Molecular Testing for Biomarker-Directed Therapy
Source: Cancer Res Commun. 2024 Oct 4;4(10):2598–609. doi: 10.1158/2767-9764.CRC-24-0321 (PMC11450693; doi:10.1158/2767-9764.CRC-24-0321)
Supplement: Supplementary Figure S7 — Gene mutations according to poverty level and RUCA codes [file crc-24-0321_supplementary_figure_s7_suppsf7.docx]

**Supplementary Figure S7. Gene mutations according to poverty level and RUCA codes. (A)** Median poverty levels for (-) and (+) gene mutation groups. **(B)** Distribution of RUCA codes among gene mutation (-) and (+) groups (numbers may add up to greater than 100 due to rounding). * *P* < .05

**
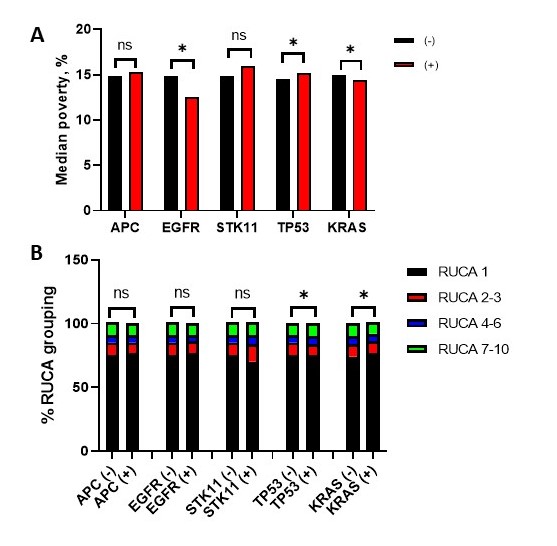
**
