# Supplementary material for: Health Disparities among Patients with Cancer Who Received Molecular Testing for Biomarker-Directed Therapy
Source: Cancer Res Commun. 2024 Oct 4;4(10):2598–609. doi: 10.1158/2767-9764.CRC-24-0321 (PMC11450693; doi:10.1158/2767-9764.CRC-24-0321)
Supplement: Supplementary Figure S8 — Comparison of overall survival among chemotherapy-treated TP53-mutated and TP53-wild type patients in Caris CODEai clinico-genomic database [file crc-24-0321_supplementary_figure_s8_suppsf8.docx]

**Supplementary Figure S8. Comparison of overall survival among chemotherapy-treated *TP53*-mutated and *TP53*-wild type patients in Caris CODEai clinico-genomic database. (A-C)** Kaplan-Meier curve analysis of *TP53*-mutated (mut) patients treated with carboplatin **(A)**, paclitaxel **(B)**, or fluorouracil **(C;** blue lines) versus patients not treated with those indicated drugs (red lines). **(D-F)** Kaplan Meier curve analysis of *TP53*-wild type (wt) patients treated with carboplatin **(D)**, paclitaxel **(E)**, or fluorouracil **(F;** blue lines) versus patients not treated with those indicated drugs (red lines). OS shown from first of treatment to last contact, based on insurance claims data.

**
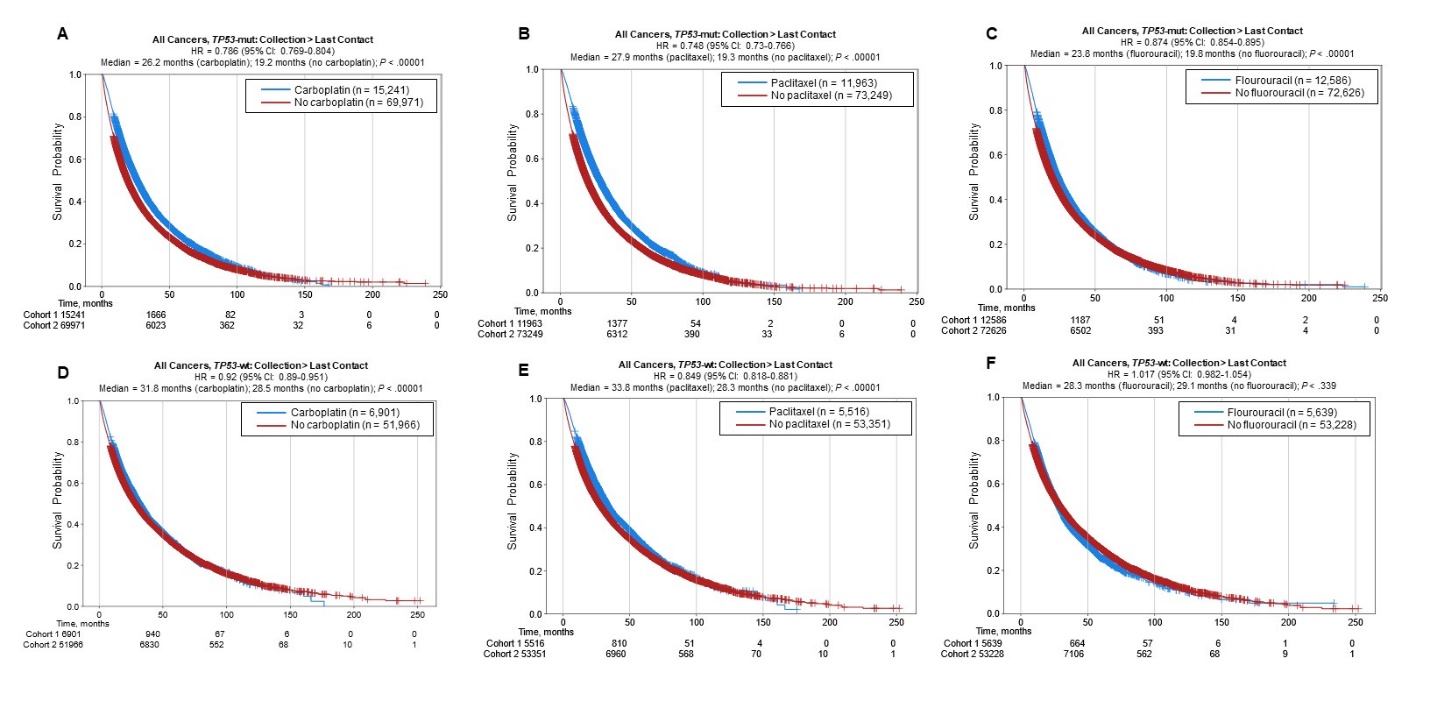
**
