# Supplementary material for: Health Disparities among Patients with Cancer Who Received Molecular Testing for Biomarker-Directed Therapy
Source: Cancer Res Commun. 2024 Oct 4;4(10):2598–609. doi: 10.1158/2767-9764.CRC-24-0321 (PMC11450693; doi:10.1158/2767-9764.CRC-24-0321)
Supplement: Supplementary Table S1 — Description of rural-urban commuting area (RUCA) codes [file crc-24-0321_supplementary_table_s1_suppst1.docx]

**Supplementary Table S1. Description of rural-urban commuting area (RUCA) codes.**

| **RUCA Code** | **Classification description** |
| --- | --- |
| **1** | Metropolitan area core: primary flow within an urbanized area (UA) |
| **2** | Metropolitan area high commuting: primary flow 30% or more to a UA |
| **3** | Metropolitan area low commuting: primary flow 10% to 30% to a UA |
| **4** | Micropolitan area core: primary flow within an urban cluster of 10,000 to 49,999 (large UC) |
| **5** | Micropolitan high commuting: primary flow 30% or more to a large UC |
| **6** | Micropolitan low commuting: primary flow 10% to 30% to a large UC |
| **7** | Small town core: primary flow within an urban cluster of 2,500 to 9,999 (small UC) |
| **8** | Small town high commuting: primary flow 30% or more to a small UC |
| **9** | Small town low commuting: primary flow 10% to 30% to a small UC |
| **10** | Rural areas: primary flow to a tract outside a UA or UC |

RUCA codes are organized on a scale from 1-10, with 1 being metropolitan and 10 being rural.
