# Supplementary material for: Health Disparities among Patients with Cancer Who Received Molecular Testing for Biomarker-Directed Therapy
Source: Cancer Res Commun. 2024 Oct 4;4(10):2598–609. doi: 10.1158/2767-9764.CRC-24-0321 (PMC11450693; doi:10.1158/2767-9764.CRC-24-0321)
Supplement: Supplementary Table S2 — COXPH model for patients with at least 6-mo overall survival [file crc-24-0321_supplementary_table_s2_suppst2.docx]

**Supplementary Table S2. COXPH model for patients with at least 6-mo overall survival.**

|  | **Overall (n = 8821)** | | **AL (n = 734)** | | **FL (n = 357)** | | **MI (n = 2176)** | | **NE (n = 688)** | | **TN (n = 3235)** | | **DC (n = 1631)** | |
| --- | --- | --- | --- | --- | --- | --- | --- | --- | --- | --- | --- | --- | --- | --- |
|  | **HR** | ***p* value** | **HR** | ***p* value** | **HR** | ***p* value** | **HR** | ***p* value** | **HR** | ***p* value** | **HR** | ***p* value** | **HR** | ***p* value** |
| **Male sex** | 1.18 | *< .001* | 1.19 | .479 | 1.18 | .730 | 1.00 | .971 | 1.03 | .799 | 1.30 | *< .001* | 1.15 | .197 |
| **White race** | 0.62 | *.021* | 0.20 | .051 | 2.77 | .443 | 0.35 | .065 | 0.71 | .697 | 0.65 | .135 | 1.00 | .993 |
| **Age (per 10 years) for non-White race** | 1.03 | .251 | 1.06 | .576 | 0.99 | .943 | 0.93 | .381 | 1.11 | .373 | 1.05 | .167 | 1.01 | .827 |
| **Age (per 10 years) for White race** | 1.11 | *< .001* | 1.34 | *< .001* | 0.78 | *.030* | 1.14 | *.008* | 1.17 | *.009* | 1.12 | *< .001* | 1.02 | .706 |
| ***APC* mut** | 0.78 | *.001* | 0.60 | .202 | 0.85 | .805 | 0.87 | .480 | 0.68 | .089 | 0.82 | .070 | 0.67 | .049 |
| ***TP53* mut** | 1.56 | *< .001* | 1.24 | .190 | 1.47 | .130 | 1.71 | *< .001* | 1.56 | *< .001* | 1.60 | *< .001* | 1.45 | *< .001* |
| ***EGFR* mut** | 0.71 | *.015* | 0.00 | .992 | Inf | .996 | 0.80 | .458 | 0.49 | .125 | 0.69 | .060 | 0.98 | .960 |
| ***STK11* mut** | 1.44 | *.001* | 3.65 | *< .001* | 9.92 | .150 | 1.72 | *.031* | 1.20 | .560 | 1.33 | .087 | 1.10 | .760 |

Cox proportional hazards regression analysis incorporating all variables for patients with at least 6-mo overall survival, broken up by site. HR = hazard ratio.
