# Supplementary material for: Health Disparities among Patients with Cancer Who Received Molecular Testing for Biomarker-Directed Therapy
Source: Cancer Res Commun. 2024 Oct 4;4(10):2598–609. doi: 10.1158/2767-9764.CRC-24-0321 (PMC11450693; doi:10.1158/2767-9764.CRC-24-0321)
Supplement: Supplementary Table S3 — COXPH model for patients with stage information [file crc-24-0321_supplementary_table_s3_suppst3.docx]

**Supplementary Table S3. COXPH model for patients with stage information.**

|  | **Overall (n = 4837)** | | **AL (n = 174)** | | **FL (n = 100)** | | **MI (n = 536)** | | **NE (n = 269)** | | **TN (n = 3010)** | | **DC (n = 748)** | |
| --- | --- | --- | --- | --- | --- | --- | --- | --- | --- | --- | --- | --- | --- | --- |
|  | **HR** | ***p* value** | **HR** | ***p* value** | **HR** | ***p* value** | **HR** | ***p* value** | **HR** | ***p* value** | **HR** | ***p* value** | **HR** | ***p* value** |
| **White race** | 0.87 | *.024* | 0.54 | *.028* | 0.71 | .574 | 1.00 | .991 | 0.79 | .505 | 0.87 | *.045* | 1.07 | .696 |
| **Age (per 10 years)** | 1.09 | *<.001* | 1.31 | *.032* | 0.86 | .471 | 1.20 | *.005* | 1.31 | *.013* | 1.09 | *.001* | 1.00 | .963 |
| **Male sex for non-White race** | 1.01 | .901 | 0.46 | .373 | 5.86 | .267 | 0.81 | .537 | 1.51 | .314 | 1.01 | .876 | 1.08 | .713 |
| **Male sex for White race** | 1.21 | *.001* | 3.25 | *.021* | 1.96 | .560 | 1.28 | .144 | 1.09 | .763 | 1.20 | *.009* | 1.07 | .702 |
| **STAGE = II** | 1.25 | *.024* | 0.88 | .801 | 0.33 | .303 | 1.76 | .063 | 0.42 | .102 | 1.39 | *.006* | 0.82 | .549 |
| **STAGE = III** | 1.45 | *< .001* | 0.65 | .264 | 1.46 | .654 | 1.18 | .563 | 1.15 | .760 | 1.61 | *< .001* | 1.43 | .237 |
| **STAGE = IV** | 1.85 | *< .001* | 1.40 | .375 | 0.24 | .160 | 2.25 | *.002* | 1.06 | .895 | 1.92 | *< .001* | 1.85 | .029 |
| ***APC* mut** | 0.80 | *.016* | 0.18 | .046 | NA | NA | 0.49 | *.033* | 0.64 | .243 | 0.86 | .171 | 0.81 | .405 |
| ***TP53* mut** | 1.58 | *< .001* | 1.60 | .074 | 1.30 | .614 | 1.33 | .051 | 1.46 | .113 | 1.64 | *< .001* | 1.69 | *< .001* |
| ***EGFR* mut** | 0.63 | *.004* | 0.33 | .311 | NA | NA | 0.39 | *.045* | 0.31 | .262 | 0.66 | *.022* | 1.26 | .664 |
| ***STK11* mut** | 1.36 | *.009* | 1.98 | .384 | NA | NA | 2.09 | *.016* | 0.97 | .951 | 1.31 | .061 | 0.98 | .951 |

Cox proportional hazards regression analysis incorporating all variables and stage, broken up by site. NA, not applicable.
